# Iodine Staining With Distance Countdown Improving the Safety for Reduction of Adverse Events: A Randomized Controlled Trial

**DOI:** 10.14309/ctg.0000000000000822

**Published:** 2025-01-21

**Authors:** Mingjia Xi, Xinyue Luo, Feifan Chen, Zhu Wang, Xue Xiao, Binyang Luo, Mo Chen, Tao Gan, Jinlin Yang, Kai Deng

**Affiliations:** 1 Department of Gastroenterology & Hepatology, West China Hospital, Sichuan University, Chengdu, Sichuan, China;; 2 Sichuan University-Oxford University Huaxi Gastrointestinal Cancer Centre, West China Hospital, Sichuan University, Chengdu, Sichuan, China;; 3 Department of Gastroenterology & Hepatology, The First People's Hospital of Longquanyi District, Chengdu, Sichuan, China;; 4 Department of Gerontology, Hospital of Chengdu Office of People's Government of Tibetan Autonomous Region, Chengdu, Sichuan, China;; 5 Department of Gerontology, Tibetan Chengdu Branch Hospital of West China Hospital, Sichuan University, Chengdu, Sichuan, China.

**Keywords:** Lugol iodine, endoscopy, esophageal cancer, adverse effects

## Abstract

**INTRODUCTION:**

Lugol chromoendoscopy (LCE) is valuable, cost-effective, and widely used in early esophageal cancer screening, yet it suffers from low compliance because of adverse events after LCE. In addition, the reflux of iodine during iodine staining in the upper esophagus brings the risk of bucking and aspiration. We introduced a new model called distance countdown (DC) aimed to reduce reflux during iodine staining in upper esophageal LCE.

**METHODS:**

In this randomized controlled trial, 204 patients were randomized into the DC and No-DC groups. The primary end point was the difference in the incidence of positive starch reagent reaction (iodine solution reflux) between the 2 groups. The secondary end points were the comparisons of the incidence of other adverse events after LCE between the 2 groups.

**RESULTS:**

The rate of iodine solution reflux was 1.0% in the DC group and 26.5% in the No-DC group (*P* < 0.001). Furthermore, the incidences of bucking between the 2 groups were 1.0% and 9.8% (*P* = 0.005). LCE satisfaction rates were 78.4% and 76.5% in the DC and No-DC groups (*P* = 0.363), respectively. Concerning symptoms after LCE, incidences of sore throat, pharyngeal discomfort or odor, bitter taste, and heartburn were also reduced in the DC group (all *P* < 0.05).

**DISCUSSION:**

Adding DC as an auxiliary effect during LCE would reduce the risk of iodine solution reflux, as well as other adverse events after LCE. Implementing this measure could be beneficial in improving the safety of LCE in early esophageal cancer screening.

## INTRODUCTION

Esophageal cancer (EC) is the seventh most frequently diagnosed cancer and the sixth leading cause of death. The 5-year survival rate in advanced EC is only 36%, whereas for early EC (EEC), it is almost 90% (1–4). Therefore, the goal of early diagnosis of EC has gradually become more important in the prevention and treatment of EC (5–7). EEC presented challenges in identifying with white light endoscopy, often accompanied by missed diagnoses due to the characteristic of subtle manifestations. Lugol chromoendoscopy (LCE) is highly effective in detecting EEC and its precursors, with a sensitivity ranging from 91% to 100% (8–10). In addition, the pink-color sign after LCE has a high specificity in identifying EEC and high-grade dysplasia (11–14). Targeted biopsy of abnormal regions after LCE has become a conventional approach in diagnosing early esophageal cancerous lesions, been recommended and widely used in clinical practice by various international clinical guidelines and consensus statements (15,16). Despite the rapid development of image enhancement endoscopy, considering that LCE reduces the difficulty for less experienced endoscopists in identifying EEC and that LCE imposes lower requirements on endoscopic equipment and still plays an indispensable role in EEC screening.

Reflux and pulmonary aspiration are common adverse events associated with upper gastrointestinal endoscopy examination (17). Studies have reported several adverse events after LCE, including retrosternal pain, chest discomfort, nausea, etc (18,19). In addition, LCE significantly increases the risk of bucking and reflux, leading to complications such as aspiration, hypoxemia, airway spasm, and laryngopharyngitis (20). Although some studies have explored the effects of lower iodine concentrations or substances such as sodium thiosulphate solution and vitamin C in alleviating adverse reactions after LCE (21–25), none have reported effective measures to reduce aspiration risk and other adverse events.

To address this gap, we introduced a new model called distance countdown (DC) and conducted this study to compare the incidence of iodine solution reflux between modified and routine methods. The secondary end point of this study was to investigate the incidence of adverse events in the 2 groups after LCE, such as esophageal iodine reflux, bucking, sore throat, pharyngeal discomfort or odor, retrosternal discomfort, heartburn, and nausea.

## METHODS

### Study design and patients

This study is a randomized controlled single-blind trial, conducted at West China Hospital from December 2022 to September 2023 and included patients scheduled to undergo esophagogastroduodenoscopy (EGD) for opportunistic screening and consented to LCE. The purpose of this study was to compare the safety of LCE groups with or without counting down the distance from incisors. This project was approved by the Ethics Committee on Biomedical Research of West China Hospital of Sichuan University and registered in the Chinese Clinical Trial Registry (ChiCTR2200065850).

Inclusion criteria are as follows: (i) patients aged at least 18 years, (ii) provided informed consent, and (iii) scheduled for general anesthesia. Exclusion criteria are as follows: (i) patients with a history of iodine allergy or organ failure, (ii) previously diagnosed with reflux esophagitis or with symptoms such as chest pain and heartburn during the week, (iii) with a history of radiotherapy or chemotherapy for head or neck malignancies, (iv) with esophageal varices, (v) pregnant or lactating women, (vi) patients at high risk of anesthesia, (vii) with esophageal lesions within 20 cm from the incisor under white light and narrow-band imaging (NBI), and (viii) patients who were not appropriate to continue this research would be eliminated.

We processed a preliminary experiment to estimate the sample size. Among the 40 patients, the reflux rate of iodine solution in groups with or without a countdown was 0.0% (0/19) and 23.8% (5/21), respectively. Using PASS15 software, we estimated that at least 180 patients were needed. All patients included were randomly assigned through randomized digital tables.

### Interventions

All patients were sedated with intravenous propofol and underwent white light endoscopy examination (Olympus CV-290) of the esophagus, stomach, and duodenum, followed by esophageal chromoendoscopy with 1.5% Lugo iodine solution sprayed with a catheter by the assistant. To ensure complete coverage of the LCE area, we prepared a 10–20 mL iodine solution per patient (the specific dosage was determined by the operator). Before LCE, the anesthesiologist elevated the patient's head to reduce the risk of aspiration.

The patients were divided into the DC group and the No-DC group. The operator performed LCE during the withdrawal of the scope from the Z line in both groups. All endoscopists were informed to stop iodine staining at 20 cm from the incisor.

**Figure 1.**
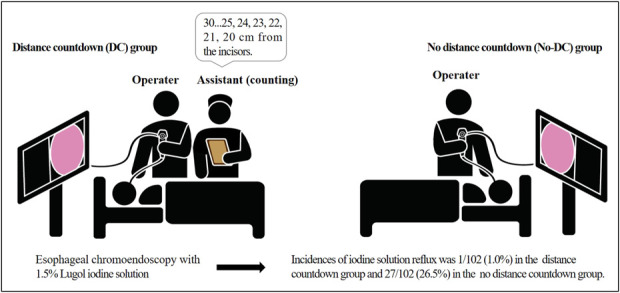
Mode pattern of Lugol chromoendoscopy in the 2 groups.

For the DC group, we assigned the assistant to help stop spraying the iodine solution. The assistant started counting when it was about 30 cm from the incisor and counted every centimeter during the LCE (Figure 1). For the No-DC group, the operator independently withdrew the scope, estimated the distance from the incisor, and instructed the assistant to spray the iodine solution at the same time. The data recorder recorded the accurate distance from which the staining stopped.

**Figure 2.**
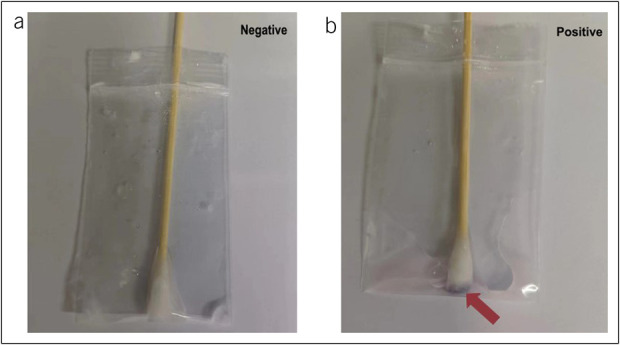
The presence of starch indicator reaction for detecting iodine solution reflux (**a** Negative, **b** Positive).

After LCE, the operator removed residual iodine solution from the stomach and then evaluated the effectiveness of LCE with good (clear boundary between the stained and faded areas), fair (distinguishable but unclear boundaries), and poor (unclear border), approximately 1–3 minutes after LCE (20). Esophageal spasm was defined as mild (no resistance when withdrawing the endoscope), moderate (slight but not significant resistance), and severe (substantial resistance). Similarly, iodine dyeing time (seconds), iodine volume (mL), iodine staining distance (cm), and bucking were recorded by the data recorder (bucking was recorded between the beginning of LCE and the end of EGD). The recorder then took a throat swab and performed a starch indicator reaction as described in a previous study (25) (Figure 2). All patients were inquired about symptoms within 1 hour after the LCE by questionnaires, and patients who felt pain were further assessed by Wong-Baker Faces Pain Scale revision.

### Data collection

Hospitalization number, age, sex, height, weight, history of smoking and drinking, comorbidity (hypertension, diabetes, coronary heart disease, heart failure, respiratory diseases, etc), operating history, and symptoms were collected by the assistant before interventions. The inspection time was defined as the interval between the time of the first and the last endoscopic images captured by the operator. The pink-color sign (positive/negative), starch indicator reaction (positive/negative), and symptoms after LCE (sore throat, chest pain, abdominal pain, pharyngeal discomfort or odor, cough, bitter taste, acid reflux, retrosternal discomfort, heartburn, nausea, chest distress or shortness of breath, bloating) were also recorded.

### Statistical analysis

We used Microsoft Office Excel to process data and Stata 17.0 software to perform statistical analysis. Quantitative data with normal distribution were represented by mean ± SD (x ± SD), and differences between groups were calculated with a *t*-test. Quantitative data with abnormal distribution were expressed as median (quartile), and the rank sum test was used to compare group differences. The categorical variable was represented by the frequency (%), and the χ^2^ test was used to compare the differences between groups. *P* value <0.05 was used as the criterion for statistical significance.

## RESULTS

### Baseline information

A total of 204 participants were included in this study, and the demographic and partial characteristics of LCE are presented in Table 1. Of these, 102 participants were randomly assigned to the DC group and the others to the No-DC group. In our study, there were 58 men (56.9%) in the DC group, with a median age of 53.00 years and a mean body mass index (BMI) of 23.28. In the No-DC group, there were 49 men (48.0%) with a median age of 55.00 years and a mean BMI of 23.83. There were no significant statistical differences between the 2 groups. The iodine usage volume in the DC group and the No-DC group were 11.52 ± 0.36 mL and 11.52 ± 0.31 mL, respectively (*P* = 0.992). In addition, there were no significant statistical differences in smoking history, drinking history, comorbidity, operating history, iodine dyeing time, and inspection time (Table 1). In addition, 2 cases of low-grade intraepithelial neoplasia and 1 case of Barrett's esophagus were detected in the DC group. 6 lesions were detected in the No-DC group, which were 4 cases of low-grade intraepithelial neoplasia, 1 case of high-grade intraepithelial neoplasia, and 1 case of Barrett's esophagus.

**Table 1.** Baseline information and LCE-related characteristics

	DC group	No-DC group	*P* value
Male	58 (56.9%)	49 (48.0%)	0.207
Age (yr)	53.00 (11.00)	55.00 (12.00)	0.078
Body mass index (kg/m^2^)	23.28 ± 0.32	23.83 ± 0.33	0.237
Smoking history			0.502
Never	75 (73.5%)	78 (76.5%)	
<10 yr	0 (0.0%)	1 (1.0%)	
≥10 yr	27 (26.5%)	23 (22.6%)	
Drinking history			0.265
Never	92 (90.2%)	84 (82.4%)	
<10 yr	1 (1.0%)	2 (2.0%)	
≥10 yr	9 (8.8%)	16 (15.7%)	
Operation history			0.491
Never	46 (45.1%)	42 (41.2%)	
Thoracic and abdominal	21 (20.6%)	17 (16.7%)	
Other	35 (34.3%)	43 (42.2%)	
Comorbidity			0.339
No	73 (71.6%)	81 (79.4%)	
Coronary heart disease	0 (0.0%)	1 (1.0%)	
Hypertension	14 (13.7%)	5 (4.9%)	
Diabetes	2 (2.0%)	2 (2.0%)	
Other disease	8 (7.8%)	8 (7.8%)	
Multiple comorbidities	5 (4.9%)	5 (4.9%)	
Iodine dyeing time (s)	17.00 (9.00)	17.00 (9.00)	0.307
Iodine volume (mL)	11.52 ± 0.36	11.52 ± 0.31	0.992
Inspection time (s)	619.00 (579.00)	707.00 (507.00)	0.572

DC, distance countdown; LCE, Lugol chromoendoscopy.

### Assessment of the safety of LCE

**Figure 3.**
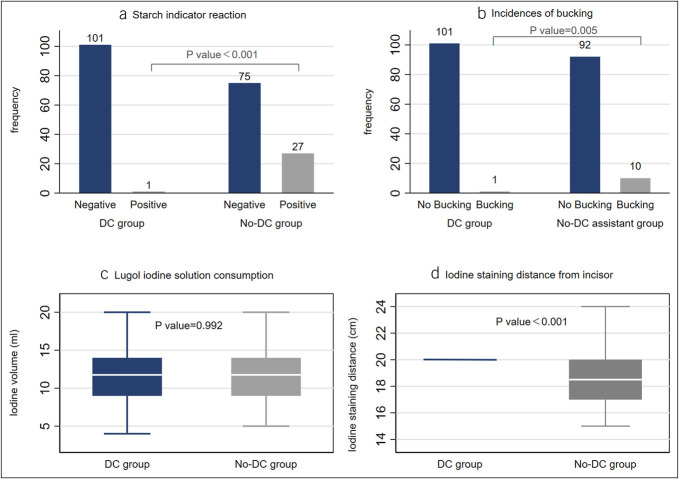
Comparison of primary outcomes between the DC and No-DC groups: (**a**) starch indicator reaction, (**b**) incidences of bucking, (**c**) Lugol iodine solution consumption, (**d**) iodine staining distance from incisor. DC, distance countdown.

We used starch indicator reaction to objectively detect Lugo iodine reflux in both groups. We also recorded the occurrence of bucking during LCE, as well as the effect of LCE assessed by endoscopists. The incidence of positive starch reagent (iodine reflux) was 1.0% and 26.5% (*P* < 0.001) in the DC and No-DC groups, respectively, and the incidence of bucking was 1.0% and 9.8% (*P* = 0.005) in the DC and No-DC groups. There was also a significant difference in LCE distance from the incisor between the DC group (20.00 [0.00]) and the No-DC group (18.50 [3.00]) (*P* < 0.001). The terminal distance in the No-DC group was shorter than the target distance, which would increase the risk of iodine solution reflux (Figure 3).

In the assessment of LCE effects, 78.43% of participants in the DC group were assessed as good, and in the No-DC group, the proportion was 76.5% (*P* = 0.363). Moreover, we recorded the assessment of esophageal spasm, and there was also no significant difference between the 2 groups (*P* = 0.431) (Table 2).

**Table 2.** Comparison of the safety and efficacy between the DC and No-DC groups during LCE

	DC group	No-DC group	*P* value
Iodine staining distance (cm)	20.00 (0.00)	18.50 (3.00)	<0.001
Esophageal spasm			0.431
Mild	35 (34.3%)	27 (26.5%)	
Moderate	35 (34.3%)	42 (41.2%)	
Severe	32 (31.4%)	33 (32.4%)	
Buck			0.005
No	101 (99.0%)	92 (90.2%)	
Yes	1 (1.0%)	10 (9.8%)	
Starch indicator reaction			<0.001
Negative	101 (99.0%)	75 (73.5%)	
Positive	1 (1.0%)	27 (26.5%)	
Iodine staining effect			0.363
Good	80 (78.4%)	79 (76.5%)	
Fair	22 (21.6%)	20 (21.6%)	
Poor	0 (0.0%)	2 (2.0%)	

DC, distance countdown; LCE, Lugol chromoendoscopy.

### Incidence of various adverse events after LCE

**Figure 4.**
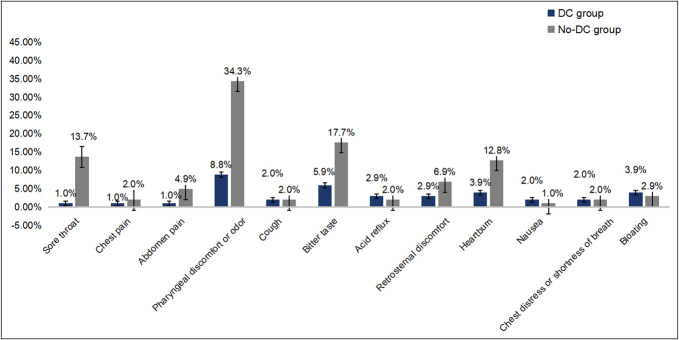
The incidences of adverse events after esophagogastroduodenoscopy. DC, distance countdown.

We investigated the symptoms of participants within 1 hour after LCE. In this study, the incidence of sore throat, pharyngeal discomfort and odor, bitter taste, and heartburn was significantly higher in the No-DC group. The sore throat rate was 1.0% (1/102) and 13.7% (14/102) in the DC and No-DC groups, respectively. The incidence of pharyngeal discomfort and odor after LCE was 8.8% (9/102) and 34.3% (35/102) (Table 3, Figure 4). For chest pain, abdominal pain, cough, acid reflux, retrosternal discomfort, nausea, chest distress and shortness of breath, and bloating, there was no significant statistical difference between the 2 groups.

**Table 3.** Incidences of LCE-related adverse events

	DC group	No-DC group	*P* value
Sore throat	1 (1.0%)	14 (13.7%)	<0.001
Chest pain	1 (1.0%)	2 (2.0%)	0.561
Abdominal pain	1 (1.0%)	5 (4.9%)	0.097
Pharyngeal discomfort or odor	9 (8.8%)	35 (34.3%)	<0.001
Cough	2 (2.0%)	2 (2.0%)	1.000
Bitter taste	6 (5.9%)	18 (17.7%)	0.009
Acid reflux	3 (2.9%)	2 (2.0%)	0.651
Retrosternal discomfort	3 (2.9%)	7 (6.9%)	0.195
Heartburn	4 (3.9%)	13 (12.8%)	0.023
Nausea	2 (2.0%)	1 (1.0%)	0.561
Chest distress or shortness of breath	2 (2.0%)	2 (2.0%)	1.000
Bloating	4 (3.9%)	3 (2.9%)	0.701

DC, distance countdown; LCE, Lugol chromoendoscopy.

### Subgroup analysis in patients with different BMI

We divided the patients into the overweight group (BMI ≥24 kg/m^2^) and the normal weight group (BMI <24 kg/m^2^), and subgroup analysis was conducted. In both the No-DC group and DC group, overweight patients included a higher proportion of male patients and had a longer smoking history (Supplementary Table 1, http://links.lww.com/CTG/B272). However, no significant difference was detected in the safety or incidences of adverse events between normal weight and overweight patients (Supplementary Tables 2 and 3, http://links.lww.com/CTG/B272).

To investigate whether overweight patients gain more benefits from DC, we compared the safety and incidences of adverse events between DC and No-DC patients, separately within the normal weight and overweight groups. DC patients comprised 51.3% (59/115) in the normal weight group, whereas they comprised 48.3% (43/89) in the overweight group (Tables 4 and 5). In both the overweight group and normal weight group, the incidence rates of positive starch reagent (iodine reflux), sore throat, and pharyngeal discomfort or odor were significantly higher in the No-DC patients when compared with DC patients (positive starch reagent: *P* < 0.001, *P* < 0.001; sore throat: *P* = 0.012, *P* = 0.008; pharyngeal discomfort or odor: *P* = 0.001, *P* = 0.004). The iodine staining distance control in the DC patients was better than that in the No-DC patients (*P* < 0.001, *P* < 0.001). In particular, DC significantly decreased the risk of bucking from 13.0% to 0.0% in overweight patients (*P* = 0.014), whereas the risk slightly reduced from 7.1% to 1.7% in the normal weight group (*P* = 0.152). In addition, in the normal weight group, DC patients showed a lower incidence of heartburn than No-DC patients (*P* = 0.021), whereas DC decreased the risk of bitter taste (*P* = 0.034) in the overweight group. No significant differences were found between the 2 groups in Lugol iodine solution consumption, iodine staining time, iodine staining effect, incidence of esophageal spasm, and risks of adverse reactions.

**Table 4.** Comparison of the safety in subgroups

	Normal weight group (n = 115)			Overweight group (n = 89)		
	DC patients (n = 59)	No-DC patients (n = 56)	*P* value	DC patients (n = 43)	No-DC patients (n = 46)	*P* value
Lugol iodine solution consumption/mL	11.56 (3.65)	11.98 (3.01)	0.501	11.45 (3.55)	10.95 (3.13)	0.484
Iodine staining time/s	16.00 (8.00)	17.00 (9.00)	0.689	18.00 (11.00)	16.50 (9.00)	0.269
Iodine staining distance/cm	20.00 (0.00)	18.50 (2.00)	<0.001	20.00 (0.00)	18.50 (3.00)	<0.001
Esophageal spasm			0.774			0.512
Mild	19 (32.2%)	15 (26.8%)		16 (37.2%)	12 (26.1%)	
Moderate	20 (33.9%)	22 (39.3%)		15 (34.9%)	20 (43.5%)	
Severe	20 (33.9%)	19 (33.9%)		12 (27.9%)	14 (30.4%)	
Buck			0.152			0.014
Yes	1 (1.7%)	4 (7.1%)		0 (0.0%)	6 (13.0%)	
No	58 (98.3%)	52 (92.9%)		43 (100.0%)	40 (87.0%)	
Starch indicator reaction			<0.001			<0.001
Negative	59 (100.0%)	44 (78.6%)		42 (97.7%)	31 (67.4%)	
Positive	0 (0.0%)	12 (21.4%)		1 (2.3%)	15 (32.6%)	
Iodine staining effect			0.475			0.513
Good	48 (81.4%)	42 (75.0%)		32 (74.4%)	36 (78.3%)	
Fair	11 (18.6%)	13 (23.2%)		11 (25.6%)	9 (19.6%)	
Poor	0 (0.0%)	1 (1.8%)		0 (0.0%)	1 (2.2%)	

DC, distance countdown.

**Table 5.** Comparison of the incidences of adverse events between subgroups

	Normal weight group (n = 115)			Overweight group (n = 89)		
	DC patients (n = 59)	No-DC patients (n = 56)	*P* value	DC patients (n = 43)	No-DC patients (n = 46)	*P* value
Sore throat	1 (1.7%)	8 (14.3%)	0.012	0 (0.0%)	7 (15.2%)	0.008
Chest pain	1 (1.7%)	1 (1.8%)	1.000	0 (0.0%)	1 (2.2%)	1.000
Abdomen pain	1 (1.7%)	4 (7.1%)	0.152	0 (0.0%)	1 (2.2%)	0.331
Pharyngeal discomfort or odor	5 (8.5%)	19 (33.9%)	0.001	4 (9.3%)	16 (34.8%)	0.004
Bitter taste	5 (8.5%)	11 (19.6%)	0.084	1 (2.3%)	7 (15.2%)	0.034
Cough	0 (0.0%)	0 (0.0%)	—	2 (4.7%)	2 (4.4%)	1.000
Acid reflux	3 (5.1%)	1 (1.8%)	0.335	0 (0.0%)	1 (2.2%)	0.331
Retrosternal discomfort	2 (3.4%)	4 (7.1%)	0.366	1 (2.3%)	3 (6.5%)	0.340
Heartburn	2 (3.4%)	9 (16.1%)	0.021	2 (4.7%)	4 (8.7%)	0.447
Chest distress or shortness of breath	0 (0.0%)	2 (3.6%)	0.143	2 (4.7%)	0 (0.0%)	0.139
Nausea	2 (3.4%)	0 (0.0%)	0.629	0 (0.0%)	1 (2.2%)	0.331
Bloating	4 (6.8%)	1 (1.8%)	0.189	0 (0.0%)	2 (4.4%)	0.167

DC, distance countdown.

## DISCUSSION

Most EC was detected at an advanced stage and had a poor prognosis because its symptoms were not typical in the early stages. Therefore, early detection of EC is crucial for reducing the associated mortality. In recent years, LCE and image enhancement endoscopy with NBI and blue light imaging have been increasingly applied in the early detection of EC (26–28). Research showed that LCE had similar sensitivities to NBI, but the specificity and accuracy of LCE were lower than that of NBI (29). Despite this, LCE is still commonly used during daily practice because it provides great convenience in identifying the border of the lesion before endoscopic submucosal resection (30). Moreover, considering its better performance in nonexpert endoscopists (27) and its economic advantages in the widespread availability of endoscopic equipment, LCE is an irreplaceable technique in the screening of EEC.

The principle of LCE is that when esophageal squamous epithelial cells become cancerous, glycogen particles in cancer cells may decrease or even disappear, and the LCE will show a light or unstained color, which can be used to show the lesion and its scope. However, due to the oxidation of iodine, many patients may suffer chest pain, heartburn, bitter taste, and so on during LCE. Several studies have concentrated on relieving discomfort after LCE and showed that 1.0%–1.5% Lugol iodine solution can minimize adverse events and, at the same time, ensure the detection rate of lesions (20,23,25). Some researches confirmed that sodium thiosulphate solution and vitamin C can also reduce adverse events after LCE (22,24). Recently, some studies have reported that LCE in the upper esophagus was at risk of reflux of iodine solution, which can further cause pharyngitis. However, it is important to conduct adequate iodine staining in the upper esophagus for the integrated screen of EEC, especially in patients with high-risk factors (31,32). Reflux and pulmonary aspiration are common during upper gastrointestinal endoscopy examination (17), but few studies focused on this. Only one study explored the risk of aspiration reflux, and the evidence quality is still poor. More research was needed to further explore this problem.

This study further explored the effect of assistant counting down distance on reducing the risk of iodine solution reflux in upper esophageal iodine staining and showed the incidence of adverse events related to LCE. In our study, the incidence of iodine solution reflux in the DC group was 1.0% but 26.5% in the No-DC group (*P* < 0.001). In addition, the assistant counting down reduced the incidence of bucking during the procedure, especially in overweight patients (1.7% vs 7.1% in the normal weight group, *P* = 0.152; 0.0% vs 13.0% in the overweight group, *P* = 0.014). For symptoms after LCE, incidences of sore throat, pharyngeal discomfort or odor, bitter taste, and heartburn differed between the DC and No-DC groups (*P* < 0.05). The LCE satisfaction rate was 78.4% in the DC group and 76.5% in the No-DC group. All patients reached the LCE criterion of 20.00 cm from the incisor, and the median distance in the No-DC group was 18.50 cm. Our study affirmed that assisting with counting down distance can effectively help achieve the ideal iodine staining distance and further reduce laryngopharyngeal reflux in LCE.

In this study, we regard that the anesthesia was a factor that rarely caused bias for the primary principle of aspiration in general anesthesia was the reduction of pharyngeal reflex. Besides, the eligibility criteria also proved a baseline balance between the 2 groups. There were also some limitations in our research. First, this study was a single-blind study. To minimize the potential bias between the groups, we used objective index, e.g., starch indicator reaction and iodine staining distance in the measurement of the primary end points. The recorder collected questionnaires on symptoms after LCE from all patients who were blinded. In addition, this study existed some confounding factors, such as the front endoscopy may also remain in iodine solution and further pollute the throat causing false-positive results in the starch indicator reaction. Although the confounding factors mentioned were difficult to avoid, they can be partially balanced through the random allocation process. Moreover, the open to assistants might influenced the research, but we adopted an objective index to verify the reflux of iodine solution, which would reduce the bias effectively. Last, we took the most commonly used LCE certification—20 cm from the incisor, there were no further data for analysis and discussion of the detection rate in upper esophageal lesions, which needs more research to explore in the future.

This study compared the effect of LCE and the incidences of adverse events in DC and No-DC groups. We affirmed that the DC group was as effective as the No-DC group during the LCE. In addition, with assistance helping to countdown distance, the incidence of iodine solution reflux in the laryngopharyngeal decreased significantly, further reducing the relative adverse events. In clinical practice, implementing this measure could be beneficial in improving the safety of LCE in EEC screening.

## CONFLICTS OF INTEREST

**Guarantor of the article:** Kai Deng, MD.

**Specific author contributions:** X.L.: collected data, performed analysis and drafted the original manuscript. M.X.: collected data, performed analysis and edited the manuscript. F.C., Z.W., X.X., B.L., M.C., T.G.: performed the study and revised manuscript. K.D. and J.Y.: conceived and designed the study and revised the manuscript.

**Financial support:** Founded by the National Natural Science Foundation of China (No. 82470536), Sichuan Science and Technology Program (22ZDYF1618 and 2022YFH0003), Supported by Chengdu Medical Research Program (No. 2022359), Teaching Fund of West China School of Medicine (HXBK-B2023052 and GSSCU2023088), the major technology application and demonstration project, Chengdu Science and Technology Bureau (2021-YF09-00050-SN), Tibet Autonomous Region Science and Technology Plan Joint Funding Project (XZ202301ZY0050G), the National Key Research and Development Program of China (2022YFC3602101 and 2022YFC3602105).

**Potential competing interests:** None to report.

**Study Highlights**

✓ Although the incidence is low, our study revealed that bucking and aspiration remain a potential risk during Lugol’s chromoendoscopy (LCE).
✓ We introduced a new approach, distance countdown (DC), which reduces the risk of iosine solution reflux and other adverse effects.
✓ The use of DC enhanced the safety of LCE, potentially improving the application of LCE in early esophageal cancer screening.

## Supplementary Material

SUPPLEMENTARY MATERIAL
