# Supplementary material for: Iodine Staining With Distance Countdown Improving the Safety for Reduction of Adverse Events: A Randomized Controlled Trial
Source: Clin Transl Gastroenterol. 2025 Jan 21;16(3):e00822. doi: 10.14309/ctg.0000000000000822 (PMC11932585; doi:10.14309/ctg.0000000000000822)
Supplement: SUPPLEMENTARY MATERIAL [file ct9-16-e00822-s001.docx]

**Supplementary Table 1. Comparison of baseline characteristics between normal weight group and overweight group**

|  | **No-DC Group** | | | **DC Group** | | |
| --- | --- | --- | --- | --- | --- | --- |
|  | **Normal weight group**  **(n=56)** | **Overweight group**  **(n=46)** | **P Value** | **Normal weight group**  **(n=59)** | **Overweight group**  **(n=43)** | **P Value** |
| **Age** | 54.96 (8.40) | 55.52 (8.69) | 0.743 | 53.95 (9.13) | 52.47 (6.83) | 0.372 |
| **Male** | 19 (33.9%) | 30 (65.2%) | 0.002 | 26 (44.1%) | 32 (74.4%) | 0.002 |
| **Smoking history** |  |  | 0.073 |  |  | 0.036 |
| never | 47 (83.9%) | 31 (67.4%) |  | 48 (81.4%) | 27 (6.8%) |  |
| < 10 years | 0 (0.0%) | 1 (2.2%) |  | 0 (0.0%) | 0 (0.0%) |  |
| > 10 years | 9 (16.1%) | 14 (30.4%) |  | 11 (18.6%) | 16 (37.2%) |  |
| **Drinking history** |  |  | 0.187 |  |  | 0.374 |
| never | 49 (87.5%) | 35 (76.1%) |  | 55 (93.2%) | 37 (86.1%) |  |
| < 10 years | 0 (0.0%) | 2 (4.4%) |  | 0 (0.0%) | 1 (2.3%) |  |
| > 10 years | 7 (12.5%) | 9 (19.6%) |  | 4 (6.8%) | 5 (11.6%) |  |

**DC: distance countdown.**

**Supplementary Table 2. Comparison of the safety between normal weight group and overweight group**

|  | **No-DC Group** | | | **DC Group** | | |
| --- | --- | --- | --- | --- | --- | --- |
|  | **Normal weight group**  **(n=56)** | **Overweight group**  **(n=46)** | **P Value** | **Normal weight group**  **(n=59)** | **Overweight group**  **(n=43)** | **P Value** |
| **Iodine staining time /s** | 17.46 (5.37) | 17.83 (6.33) | 0.755 | 18.54 (6.47) | 19.7 (7.50) | 0.407 |
| **Iodine staining effect** |  |  | 0.905 |  |  | 0.400 |
| good | 42 (75.0%) | 36 (78.3%) |  | 48 (81.4%) | 32 (74.4%) |  |
| fair | 13 (23.2%) | 9 (19.6%) |  | 11 (18.6%) | 11 (25.6%) |  |
| poor | 1 (1.8%) | 1 (2.2%) |  | 0 (0.0%) | 0 (0.0%) |  |
| **Iodine solution consumption /mL** | 11.982 (3.01) | 10.957 (3.13) | 0.096 | 11.559 (3.65) | 11.453 (3.55) | 0.884 |
| **Iodine staining distance /cm** | 18.11 (1.58) | 18.54 (2.25) | 0.270 | 20 (0.00) | 20 (0.00) |  |
| **Starch indicator reaction** |  |  | 0.203 |  |  | 0.422 |
| negative | 44 (78.6%) | 31 (67.4%) |  | 59 (100.0%) | 42 (97.7%) |  |
| positive | 12 (21.4%) | 15 (32.6%) |  | 0 (0.0%) | 1 (2.3%) |  |
| **Buck** |  |  | 0.508 |  |  | 1.000 |
| yes | 4 (7.1%) | 6 (13.0%) |  | 1 (1.7%) | 0 (0.0%) |  |
| no | 52 (92.9%) | 40 (87.0%) |  | 58 (98.3%) | 43 (100.0%) |  |
| **Esophageal spasm** |  |  | 0.907 |  |  | 0.823 |
| mild | 15 (26.8%) | 12 (26.1%) |  | 19 (32.2%) | 16 (37.2%) |  |
| moderate | 22 (39.3%) | 20 (43.5%) |  | 20 (33.9%) | 15 (34.9%) |  |
| severe | 19 (33.9%) | 14 (30.4%) |  | 20 (33.9%) | 12 (27.9%) |  |

**DC: distance countdown.**

**Supplementary Table 3. Comparison of the incidences of adverse events between normal weight group and overweight group**

|  | **No-DC Group** | | | **DC Group** | | |
| --- | --- | --- | --- | --- | --- | --- |
|  | **Normal weight group (n=56)** | **Overweight group (n=46)** | **P Value** | **Normal weight group (n=59)** | **Overweight group (n=43)** | **P Value** |
| **Sore throat** | 8 (14.3%) | 7 (15.2%) | 0.895 | 1 (.7%) | 0 (0.0%) | 1.000 |
| **Chest pain** | 1 (1.8%) | 1 (2.2%) | 1.000 | 1 (1.7%) | 0 (0.0%) | 1.000 |
| **Abdomen pain** | 4 (7.1%) | 1 (2.2%) | 0.487 | 1 (1.7%) | 0 (0.0%) | 1.000 |
| **Pharyngeal discomfort or odor** | 19 (33.9%) | 16 (34.8%) | 0.928 | 5 (8.5%) | 4 (9.3%) | 1.000 |
| **Bitter taste** | 11 (19.6%) | 7 (15.2%) | 0.560 | 5 (8.5%) | 1 (2.3%) | 0.380 |
| **Cough** | 0 (0.0%) | 2 (4.4%) | 0.201 | 0 (0.0%) | 2 (4.7%) | 0.175 |
| **Acid reflux** | 1 (1.8%) | 1 (2.2%) | 1.000 | 3 (5.1%) | 0 (0.0%) | 0.261 |
| **Retrosternal discomfort** | 4 (7.1%) | 3 (6.5%) | 1.000 | 2 (3.4%) | 1 (2.3%) | 1.000 |
| **Heartburn** | 9 (16.1%) | 4 (8.7%) | 0.416 | 2 (3.4%) | 2 (4.7%) | 1.000 |
| **Chest distress or shortness of breath** | 2 (3.6%) | 0 (0.0%) | 0.500 | 0 (0.0%) | 2 (4.7%) | 0.175 |
| **Nausea** | 0 (0.0%) | 1 (2.2%) | 0.451 | 2 (3.4%) | 0 (0.0%) | 0.507 |
| **Bloating** | 1 (1.8%) | 2 (4.4%) | 0.862 | 4 (6.8%) | 0 (0.0%) | 0.136 |

**DC: distance countdown.**
